# Reform of the Medical Acts

**Published:** 1900-03-10

**Authors:** 


### REFORM OF THE MEDICAL ACTS.

The decision of Mr. Paul Taylor at the North London Police court in the Alabone case may be taken, according to one's taste, either as a heavy blow and great discouragement to the medical profession, or as a proof positive that in the interests of the public the Medical Acts ought to be amended. The facts as given in the *Times* report are abundantly simple. Mr. Alabone, the defendant, was at one time a member of the Royal College of Surgeons of England, and as such he was registered under the Act as a medical practitioner. In 1886, however, the college removed his name from its list of members for "disgraceful conduct," and, that decision being communicated to the General Medical Council, the name was erased from the medical register. He, however, continued to practice, and described himself as being "M.D. Bellevue College, M.D. Phil., U.S.A., D.Sc., F.R.M.S., Ex-M.R.C.S.Eng." Hence he was summoned for that he did wilfully and falsely pretend to be a doctor of medicine, and did wilfully and falsely take and use titles and descriptions implying that he was recognised by law as a practitioner of medicine. After a lengthy hearing the magistrate, Mr. Paul Taylor, said that the suggestion of the prosecution seemed to be that because the defendant said that he was an M.D. of Bellevue College, and an M.D. of Philadelphia, it implied that he was an M.D. of the Bellevue College, New York, and of the Jefferson Medical School, Philadelphia, both of which degrees entitled the holder to registration in England. This assumption to his mind was wrong. The defendant gave the origin of his degrees, and furthermore said that he was an ex-member of the College of Surgeons. That being so, he found, as a fact, that the defendant described himself accurately as that which he was without any intention of misleading the public. The case was dismissed, and the magistrate refused to state a case for the higher court.

Thus comes to an end another attempt on the part of the General Medical Council to enforce the obvious meaning and intention of the Medical Act, and we have another illustration of the way in which the intention of an Act of Parliament may be entirely frustrated by the blundering of those who are responsible for its phraseology. No one can doubt that the object with which the Medical Act of 1858 was passed was to enable the ignorant to distinguish between those who are qualified as medical practitioners and those who are not. For this purpose it was enacted that any person who "wilfully and falsely" pretends to be, or takes or uses the name or title of a physician, doctor of medicine, &c., &c., or any name, title, addition, or description implying that he is a registered person, or that he is recognised by law as a physician, &c., &c., may be fined a sum not exceeding £20. So far as the object of the Act was concerned there was not the slightest reason or excuse for inserting the above word "falsely." The very object of the Act was to place the use of medical titles under strict control, and to prevent the possessors of any degrees or diplomas except such as were approved of and registered by the Medical Council from so using their titles as to imply that they were qualified medical practitioners. Whether such diplomas were, in fact, possessed or not was a matter of indifference; the intention was to prevent their being used unless they were registered, and so far as this intention was concerned the word "falsely" merely complicated matters. It has, indeed, so complicated matters as to render it extremely difficult to obtain a conviction if a man can prove that he really possesses the degree which he displays, however unregisterable it may be. There the word is, however, and unfortunately the magistrates persist in holding that the term "falsely" applies to the taking and using of the title, and not to the implication that the person so using it is recognised by law as a practitioner in medicine.

We must presume that the magisterial interpretation is the correct one, but the result is that, so far as carrying out its preamble is concerned, the Act is not worth the paper it is written upon. We cannot quarrel with the decision, but we maintain that the very fact that such a decision should be possible is proof that the Act ought to be revised as soon as possible. The Act was passed with the definite object of preventing medical titles being used in such a way as to mislead the public. In consequence of its stupid wording it absolutely fails to do so. The public, then, may fairly join in demanding that so inept a law should be strengthened in such a manner as to give them in reality the protection it professes to afford.

